# First Asp-2078-Gly Mutation Conferring Resistance to Different ACCase Inhibitors in a *Polypogon fugax* Population from China

**DOI:** 10.3390/ijms24010528

**Published:** 2022-12-28

**Authors:** Bocheng Mo, Wen Chen, Sifen He, Haozhe Liu, Lianyang Bai, Lang Pan

**Affiliations:** College of Plant Protection, Hunan Agricultural University, No. 1, Nongda Road, Furong District, Changsha 410128, China

**Keywords:** acetyl-CoA carboxylase (ACCase), quizalofop-p-ethyl, *Polypogon fugax*, target-site resistance (TSR), non-target-site resistance (NTSR)

## Abstract

Asia minor bluegrass (*Polypogon fugax*) is a common and problematic weed throughout China. *P. fugax* that is often controlled by acetyl-CoA carboxylase (ACCase) inhibitors in canola fields. Herein, we confirmed a *P. fugax* population (R) showing resistance to all ACCase inhibitors tested with resistance indexes ranging from 5.4–18.4. We further investigated the resistance mechanisms of this R population. Molecular analyses revealed that an amino acid mutation (Asp-2078-Gly) was present in the R population by comparing *ACCase* gene sequences of the sensitive population (S). In addition, differences in susceptibility between the R and S population were unlikely to be related to herbicide metabolism. Furthermore, a new derived cleaved amplified polymorphic sequence (dCAPS) method was developed for detecting the Asp-2078-Gly mutation in *P. fugax* efficiently. We found that 93.75% of plants in the R population carried the Asp-2078-Gly mutation, and all the herbicide-resistant phenotype of this R population is inseparable from this mutation. This is the first report of cross resistance to ACCase inhibitors conferred by the Asp-2078-Gly target-site mutation in *P. fug*ax. The research suggested the urgent need to improve the diversity of weed management practices to prevent the widespread evolution of herbicide resistance in *P. fug*ax in China.

## 1. Introduction

In plants, acetyl-CoA carboxylase (ACCase; EC 6.4.1.2) catalyzes the carboxylation of acetyl-CoA to malonyl-CoA, which is the first step in fatty acid synthesis [1]. Plant fatty acid biosynthesis is a crucial process in the formation of cellular membranes, plant lipids, or their metabolic derivatives and surface layers [2]. Aryloxyphenoxypropionates (APP), cyclohexanediones (CHD), and phenylpyrazoline (PPZ) are three chemically distinct classes of ACCase-inhibitors [3]. The mode of action of these three classes is similar by inhibiting ACCase, mainly blocking de novo fatty acid synthesis and eventually causing plant death. However, due to massive over-reliance on ACCase inhibitors, a total of 49 weeds have currently evolved resistance to ACCase inhibitors since the first case of ACCase inhibitor resistance was reported in 1982 in ryegrass [4].

The resistance mechanism of weeds to ACCase-inhibitors involves mainly target-site resistance (TSR) and non-target-site resistance (NTSR) [5]. TSR is resulted from the conformational changes of herbicide target protein due to amino acid substitution. The conformational changes led to the decrease of herbicide sensitivity to target enzymes [5]. In general, there is a high probability of mutation at or near the binding site of the herbicide and target protein [6]. To date, *ACCase* gene mutations at the codon position 1781, 1999, 2027, 2041, 2078, 2088 and 2096 have been found to confer broader resistance to multiple classes of ACCase inhibitors in several grass weeds, such as Japanese Foxtail (*Alopecurus japonicus*) [7], Chinese sprangletop (*Leptochloa chinensis* (L.) Nees) [8], American sloughgrass (*Beckmannia syzigachne* Steud) [9,10] and ryegrass (*Lolium rigidum*) [11]. NTSR mechanisms usually include reduced herbicide penetration and herbicide translocation, and enhanced toxophores metabolism, or herbicide sequestration [5,12,13]. In most cases, NTSR to ACCase-inhibitors results from enhanced metabolism [5], in which cytochrome P450 (CYP450) and glutathione S-transferase (GST) genes have been reported to enhance the weed’s ability to metabolize ACCase inhibitors [14,15].

Asia minor bluegrass (*Polypogon fugax*), an annual hexaploid weed, often invades into farmland, vegetable nurseries and even in the urban green belt [16]. Recently *P. fugax* has become the dominant weed in many areas of canola (*Brassica napus L*.) and wheat (*Triticum aestivum L.*) fields in China due to its wide environmental adaptability and strong competitiveness, leading to severe crop yield losses [16,17]. Historically, *P. fugax* in canola fields and wheat fields were effectively controlled by the ACCase inhibitors quizalofop-p-ethyl at a dose of 52.5 g a.i. ha^−1^ and fenoxaprop-p-ethyl at a dose of 62.1 g a.i. ha^−1^, respectively. However, over-reliance on ACCase inhibitors to control *P. fugax* [17,18] has resulted in a growing number of *P. fugax* populations evolving resistance to ACCase inhibitors in China [17,18,19,20], which makes the control of this weed more challenging.

Resistance to ACCase inhibitors in *P. fugax* was first reported in 2014, when the Ile-2041-Asn mutation of the *ACCase* gene in the *P. fugax* population conferred resistance to APP herbicides, but not to CHD and DEN herbicides [19]. A recent study had indicated that the Trp-1999-Ser mutation and CYP450-involved metabolism were very likely responsible for the high-level resistance to fenoxaprop-p-ethyl and pinoxaden in a *P. fugax* population AHHY [18]. Moreover, our previous research confirmed that quizalofop-p-ethyl resistance in a non-target-site resistant *P. fugax* population was likely to be GST-induced [17]. However, progress on uncovering the resistance mechanisms in *P. fugax* has so far been slow, and most amino acid substitutions at the known ACCase mutation sites are not revealed in this weed species. Resistance to ACCase inhibitors in *P. fugax* has resulted in failures for weed management, thereby causing an increasing threat to current food security. It is urgent to decipher the resistance mechanism(s) and predict the cross-resistance patterns, which helps to design effective strategies for management in *P. fugax* [21]. In this study, a putative quizalofop-p-ethyl-resistant populations were collected from canola fields in Sichuan provinces, China. The objectives of this study were to (1) determine the resistance level to quizalofop-p-ethyl in the putative resistant *P. fugax* populations; (2) confirm the cross-resistance pattern to other herbicides; (3) investigate the TSR and NTSR mechanisms; (4) develop a derived cleaved amplified polymorphic sequence (dCAPS) protocol for effective detection of the specific mutation frequency in the resistant population.

## 2. Results

### 2.1. Quizalofop-p-Ethyl Resistance

As expected, dose-response confirmed that the S population was susceptible to quizalofop-p-ethyl, and the R population showed resistance to quizalofop-p-ethyl (Table 1, Figure S1). The GR_50_ values for the R and S populations were 102.05 and 9.37 g a.i. ha^−1^, respectively. Based on the RI value, the R *P. fugax* population was approximately 11-fold resistant to quizalofop-p-ethyl (Table 1, Figure S1). PBO, malathion and NBD-CI pre-treatment did not significantly affect the quizalofop-p-ethyl susceptibility with the GR_50_ values ranging from 95.41 to 100.20 g a.i. ha^−1^ (*p* > 0.05) for the R population (Table 1, Figure S2). The GR_50_ values for the S population of treatment with PBO, malathion and NBD-CI plus quizalofop-p-ethyl were 9.85, 8.80 and 9.59 g a.i. ha^−1^, respectively, similar to that of quizalofop-p-ethyl treatment alone (9.37 a.i. ha^−1^) (*t*-test, *p* > 0.05) (Table 1, Figure S2). Thus, the cytochrome P450 inhibitors PBO and malathion, and GST inhibitor NBD-CI could not reverse quizalofop-p-ethyl resistance in the R population (Table 1).

### 2.2. Dose-Response to Other Herbicides

The R population showed different resistance levels to all tested ACCase inhibitors (Table 2, Figure S3A–H). The R population has evolved a high-level resistance to four APP herbicides (including haloxyfop-R-methyl, fenoxaprop-P-ethyl, metamifop and cyhalofop-butyl) with the RI over 10 (Table 2, Figure S4). The R population was also resistant to clodinafop-propargyl, sethoxydim, pinoxaden and clethodim with RI of 9.07, 8.82, 5.46 and 6.54, respectively (Table 2). The GR_50_ of R population to quizalofop-p-ethyl, haloxyfop-R-methyl, fenoxaprop-P-ethyl, metamifop and cyhalofop-butyl were much higher than the corresponding recommended field rates. However, the results proved that the R population has no multiple-resistance to two ALS inhibitors mesosulfuron-methyl and pyroxsulam (*p* > 0.05) (Table 2, Figure S5).

### 2.3. Identification of ACCase Mutation

**Figure 1:**
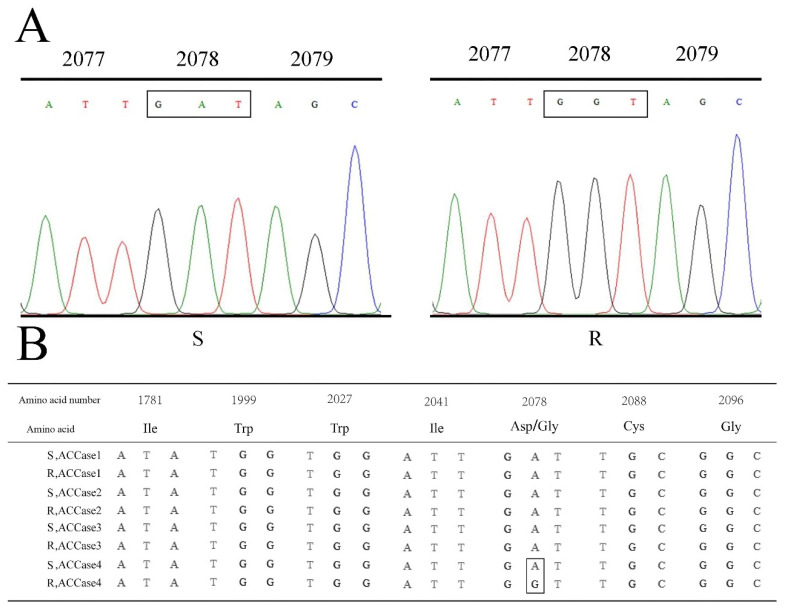
Comparison of sequencing results between R and S population: (**A**) GAT for Asp (left) in the S population and GGT for Gly (right) in the R population at codon position 2078. (**B**) Sequence alignment of four ACCase copy genes of R and S populations at 7 amino acid positions related to resistance and the wireframe reveals the mutation from Asp to Gly. R, the resistant population; S, the sensitive population.

A 1437 bp PCR fragment, including all known resistance-related amino acid substitutions (from positions 1656 to 2134), encoded 479 amino acids. Sequence alignment showed that no non-synonymous mutations were found in the four ACCase genes between the R and S population. In comparison with the reported *P. fugax* sequence of ACCase1–4 [17], all surviving plants in the R population were found to hold a GAT-to-GGT mutation at the codon position 2078 (Asp-2078-Gly) in *ACCase4* (Figure 1).

### 2.4. Determination of Quizalofop-p-Ethyl Residual in P. fugax

The retention times of quizalofop-p-ethyl were around 19.4 min (Figure S6), and the determination coefficient (R^2^) of the linear curve was 0.9999. According to the results, quizalofop-p-ethyl residual in both R and S *P. fugax* population decreased with increasing time.

**Figure 2:**
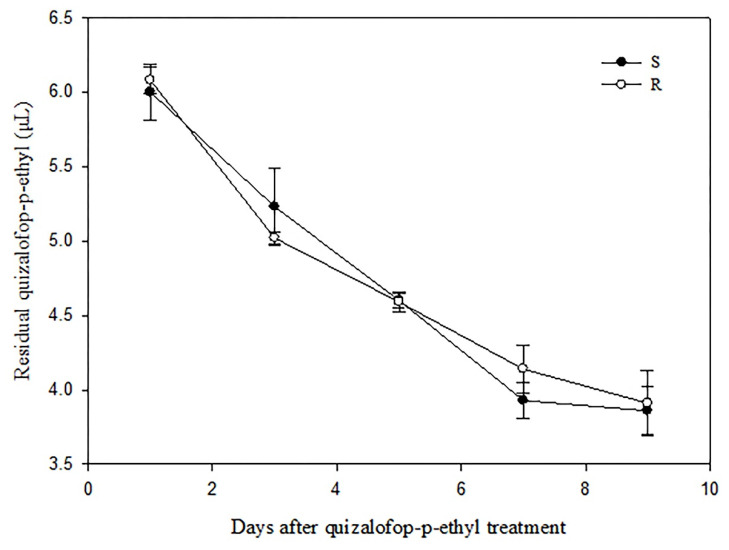
The quizalofop-p-ethyl residual in R and S *Polypogon fugax* populations 1, 3, 5, 7 and 9 days after quizalofop-p-ethyl treatment.

After quizalofop-p-ethyl treatment, quizalofop-p-ethyl present in the R and S population was confirmed to be quickly reduced from 6.08 to 5.02 μg and 6.00 to 5.23 μg from 1 to 3 day, respectively. In contrast, from the 7 to 9 days after treatment, the quizalofop-p-ethyl residual reduced slowly in these two populations, decreasing by 0.23 μg and 0.07 μg, respectively. However, quizalofop-p-ethyl in R and S plants displayed no significant differences after treatment (*t*-test, *p* > 0.05) (Figure 2).

### 2.5. The Correlation between Phenotypes and Genotypes of P. fugax

**Figure 3:**
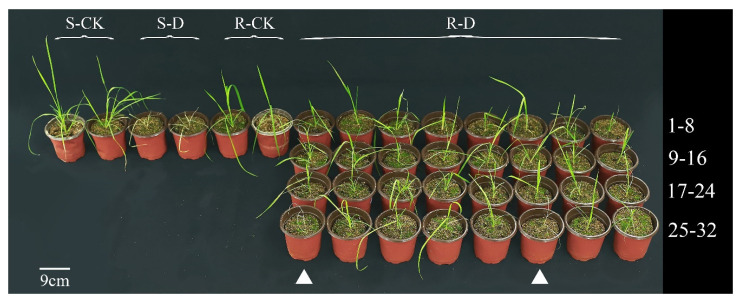
Growth effects of 32 R plants and 2 S plants used for dCAPS markers 21 days after quizalofop-p-ethyl application. Notes: S-CK, for S plants with treatment of water; S-D, for S plants with treatment of quizalofop-p-ethyl; R-CK, for R plants with treatment of water; R-D, for R plants with treatment of quizalofop-p-ethyl; 1–32, numbering of R plants. Two dead plants are marked with white triangles in the R population.

**Figure 4:**
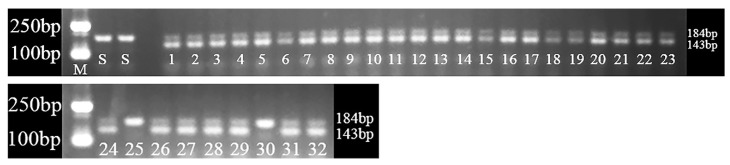
Electrophoretic patterns obtained with derived cleaved polymorphic amplified sequence (dCAPS) markers from 32 R *Polypogon fugax* plants and 2 S *Polypogon fugax* plants. Notes: M: DL2000 DNA Marker; S, sensitive plants; 1–32, numbering of resistant plants.

Quizalofop-p-ethyl treatment was applied to determine the sensitivity of the R and S populations, and each plant was genotyped using the dCAPS method. In the S populations, two single plants did not survive treatment and were confirmed to have no mutation (Figure 3 and Figure 4). However, of the 32 single plants tested in the R populations, 30 plants surviving the quizalofop-p-ethyl treatment contained the D2078G mutation (Figure 3 and Figure 4), while two dead plants (Figure 4, numbers 25 and 30) did not have the D2078G mutation. Meanwhile, 93.75% of quizalofop-p-ethyl-resistant plants (30 out of 32) in the R population showed digested mutant-type nucleotides/bands and wild-type nucleotides/bands, indicating heterozygous resistance at the position 2078 (Figure 4). These results confirmed the accuracy of this dCAPS method, and the resistance phenotype to quizalofop-p-ethyl is inseparable from the Asp-2078-Gly target-site mutation in the R population.

## 3. Discussion

Quizalofop-p-ethyl has been introduced to the market for weed control in croplands for decades. However, the long-term over-reliance has resulted in the resistance evolution to this herbicide. Thus far, seven weed species have been reported to evolve resistance to quizalofop-p-ethyl including *P. fugax* [17,19,20], *B. syzigachne* [9,10], Barnyard-Grass (*Echinochloa crusgalli* (L.) Beauv.) [22,23], Italian Ryegrass [24], *Alopecurus aequalis* Sobol [25,26], Keng Stiffgrass (*Sclerochloa kengiana*) [27] and Crabgrass (*Digitaria sanguinalis*) [4] in China. Therefore, this is a serious indication that weed management needs multiple measures than just herbicide application alone.

*P. fugax* is a troublesome weed that occurs in wheat and canola fields throughout China [18], and four ACCase copy genes were found in this polyploid weed species. In fact, target-site resistance mutation(s) occurred in any allele encoding herbicide target enzymes that can evolve herbicide resistance in weeds [6]. However, specific resistance mutation(s) might have a preference for herbicide-target isozymes. In *P. fugax*, even though a total of four ACCase copy genes (*ACCase1*, *ACCase2*, *ACCase3* and *ACCase4*) were found, only the Asp-2078-Gly substitution in *ACCase4* allele (Figure 1B) and Trp-1999-Ser mutation only in the *ACCase2* allele [18] can evolve herbicide resistance. This phenomenon regarding the ACCase preference of different resistant mutation was also observed in the polyploid species Wild Oat (*Avena fatua*) [28]. Two point mutations (1781 and 2088) were found in *ACCase1;1*/*ACCase1;2* alleles, whereas the 2078 mutation clearly occurred in *ACCase1;3*. Besides, ALS preference also existed in *Descurainia sophia* L. and *Monochoria vaginalis* populations with four ALS isozymes, and it was clearly found that most resistant mutations were biased toward specific ALS alleles [29,30]. On the whole, the Asp-to-Gly mutation at the 2078 position found only in the *P. fugax* ACCase4 gene might be resulting from the preference of this mutation, and additional experiments are required to understand the evolutionary strategies of herbicide resistance in polyploid weed species.

In addition, a new derived cleaved amplified polymorphic sequence (dCAPS) method was developed for efficiently detecting the Asp-2078-Gly mutation in *P. fugax*. In the present study, we found that the Asp-2078-Gly mutation occurred at a high frequency in this R population, and all resistant individuals were detected to be heterozygotes with wild-type bands using dCAPS markers. As several homologous ACCase genes were amplified by the designed primers in polyploid weed species, dCAPS assay is difficult to distinguish allelic heterozygosity from homoeologous heterozygotes [28]. The dCAPS markers analysis will not show wild-type nucleotides only when the same mutations are found in a homozygous state among all homologues [28]. *P. fugax* is a hexaploid (2n = 6x = 42) weed species holding four copies of the plastidic ACCase gene [18]. Similar results were also found in tetraploid *A. japonicus* and hexaploid *A. fatua* [28,31].

Asp-to-Gly was the first demonstrated mutation at the codon position 2078 of ACCase in *A. myosuroides*, and this mutation was successively detected in various weed species [32,33,34,35,36,37]. However, this is the first time the Asp-2078-Gly mutation in *P. fugax* conferring quizalofop-p-ethyl resistance has been documented. In addition, the R population showed cross-resistance to all three classes of ACCase inhibitors (Table 2, Figure S3). Although the Asp-2078-Gly belonging to non-active sites had no direct contact with the herbicide, this point mutation was found to be associated with the conformational change of binding pocket and the hydrogen-bonding interactions [38], leading to resistance to APP, CHD and PPZ herbicides.

In the study, it was noted that the quizalofop-p-ethyl-resistant *P. fugax* was also resistant to the CHD herbicides clethodim. The resistance patterns endowed by the Asp-2078-Gly mutation have been inconsistent in various species. For instance, the resistant Amazon sprangletop [*Leptochloa panicoides* (J. Presl) Hitchc.] population holding the Asp-2078-Gly substitution was found not to be resistant to clethodim [39]. In addition, the R population with the Asp-2078-Gly mutation showed a higher resistance level to APP herbicides than CHD and PPZ herbicides (Table 2, Figure 1), compared to other weed species like Japanese foxtail [40], *Phalaris paradoxa* [34] and Wild Oat (*Avena fatua*) [35].

The different resistance patterns and levels in resistant weed species could be caused by (1) specific amino acid changes; (2) different weed species [41]; (3) zygosity of the resistance allele [5]; (4) NTSR mechanisms co-evolution [10]. Specific amino acid changes can affect the pattern and level of resistance to ACCase inhibitors. For example, the Asp-2078-Gly mutation conferred metamifop resistance, while Asp-2078-Glu did not in the resistant barnyardgrass (*Echinochloa crus-galli*) AXXZ-2 population [37], causing the unique cross-resistance pattern. However, the Asp-2078-Gly mutation conferred either high or no clethodim resistance in *P. fugax* (current research) and *L. panicoides* [39], implying no absolute link between specific target-site mutations and clethodim resistance. The varied resistance to ACCase inhibitors could be caused by different weed species, although their resistance mechanisms involve the same target-site mutation [5]. For instance, the Ile-1781-Leu mutation conferred differences in resistance and non-resistance to clethodim in *Alopecurus aequalis* Sobol biotypes [42] and *A. myosuroides* Huds biotypes [33], respectively. Differences in clethodim resistance occurred frequently in different weed species, which may be related to the conformational change caused by the same amino acid mutation in specific weed species, resulting in a different affinity of clethodim to the binding pocket.

In addition, the zygosity of the resistance allele involved in TSR may play a key role in the resistance level. It was reported that heterozygous biotypes were less resistant to clodinafop-propargyl than homozygous biotypes in a *Lolium* spp. population carrying the Ile-2041-Val mutation [43]. Multiple susceptible alleles may dilute the resistance level conferred by one/several mutant alleles [28,44]. This may explain that one mutant allele in two copies of the ACCase gene conferred a higher resistance to ACCase inhibitors in Japanese foxtail [40] than that observed in four copies *P. fugax* in the current study. Indeed, intervention of NTSR mechanisms also caused changes in the resistance level to ACCase inhibitors [10]. For instance, NTSR mechanisms increased the resistance level to fenoxaprop-P-ethyl by at least 55% in a *P. fugax* population carrying the Trp-1999-Ser mutation [18]. In a perennial ryegrass (*Lolium perenne* L.) population with the Ile-2041-Val mutation, resistance mechanisms involving CYP450 could increase the resistance levels to pinoxaden from 9.7-fold to 41.4-fold [45].

In recent years, increasing cases reported that weed species evolved both TSR and NTSR to ACCase-inhibitor [18,29,46,47]. This phenomenon will be more complicated for the management of ACCase-inhibitor resistant weed populations. However, studies on the co-evolution of NTSR mechanisms in weed species carrying mutations at the 2078 position of ACCase had often been overlooked in previous reports. As the most common NTSR mechanism [5], enhanced herbicide metabolism was further explored in our R *P. fugax* population. In this study, no significant difference for quizalofop-p-ethyl residual was observed in the R and S population after quizalofop-p-ethyl treatment. In addition, all three inhibitors, including two CYP450 inhibitors and one GST inhibitor, did not increase sensitivity to quizalofop-p-ethyl in the R population. Consequently, resistance to quizalofop-p-ethyl is unlikely to be caused by enhanced herbicide metabolism, especially the involvement of CYP450 and GST. Overall, compared with other weed species with the Asp-2078-Gly mutation, the differential response to ACCase inhibitors in the *P. fugax* R population may be only due to the specific weed populations and zygosity of the resistance allele.

## 4. Materials and Methods

### 4.1. Plant Materials

In May 2017, seeds of the putative resistant (R) *P. fugax* population were collected in a canola field at Chongzhou City in Sichuan, China (30.68° N, 103.56° E). Seeds from 50 mature plants were collected randomly and saved in envelopes at room temperature. The *P. fugax* population SC-S was used as a standard sensitive (S) population in this study, which had never been applicated by herbicides [17].

### 4.2. Whole-Plant Dose-Response Bioassay

Whole-plant dose-response bioassay was carried out in a glasshouse during the normal growing season (September to December). Seeds for each population were sowed (20 plants per pot) in a single plastic pot filled with potting mix. When the seedlings of *P. fugax* were grown to the 3–4 leaf stage, they were sprayed with quizalofop-p-ethyl, or the GST inhibitor 4-chloro7-nitrobenzoxadiazole (NBD-Cl) (Sigma, Beijing, China) plus quizalofop-p-ethyl (Tianrun Co., Ltd,, Binzhou, China), or the cytochrome P450 inhibitor malathion (Sigma, Beijing, China) and piperonyl butoxide (PBO) (Sigma, Beijing, China) plus quizalofop-p-ethyl, using a moving nozzle cabinet sprayer (Tianjin Labatory Instrument Co., Ltd, Tianjin, China) equipped with a TP6501E flat fan nozzle delivering 372 L ha^−1^. Malathion (1000 g a.i. ha^−1^), NBD-Cl (270 g a.i. ha^−1^) and PBO (4200 g a.i. ha^−1^) were applied 2, 48 and 1 h, respectively, prior to quizalofop-p-ethyl application [16,17]. To characterize the cross-resistant patterns of the quizalofop-p-ethyl-resistant *P. fugax* population, eight other ACCase inhibitors (including five APPs, two CHDs and one PPZ) and two ALS inhibitors were applied when the plants grew to the 3–4 leaf stage. The doses of herbicides were listed in Table 3. As total biomass of the aboveground part is a sensitive indicator, three weeks after herbicide application, the fresh weight of the aboveground part was recorded. The experiment was conducted twice with three replicates per herbicide treatment.

### 4.3. Identification of the P. fugax Plastidic ACCase Gene Mutation

Nine R plants surviving the quizalofop-p-ethyl treatment at field-recommended rate (52.5 g a.i. ha^−1^) were obtained for identification of ACCase gene mutation. Total DNA was extracted from fresh leaf tissue using the PlantGenomic DNA Kit (TiangenBiotech Co., Ltd, Beijing, China). A pair of published primers was used to amplify the *P. fugax* plastidic ACCase CT domain gene fragment containing the seven site mutations [18]. The PCR system and procedure were performed as described [17]. The amplified PCR products were purified and then cloned into pClone007 Versatile Simple Vector (Tsingke, Beijing, China). Plasmids containing the correctly inserted fragment were sequenced bi-directionally by a sequencing company (Tsingke, Beijing, China). At least 16 clones of each plant were sequenced and compared with the four documented *P. fugax* ACCase copy genes (Accession number MK359055 to MK359058), respectively.

### 4.4. Detection of Quizalofop-p-Ethyl Residual in R and S P. fugax Populations

To compare the difference of quizalofop-p-ethyl residual in R and S *P. fugax* populations, R and S seedlings were grown in the conditions as described above. Total 20 μL quizalofop-p-ethyl solution with a concentration of 50 mg·L^−1^ (methyl alcohol dilution) was applied to two leaves of a single plant at 4–5 leaf stage by micropipettes. Ten pots (10 plants per pot) were treated by quizalofop-p-ethyl in the R and S *P. fugax* population. The aboveground parts were harvested for quizalofop-p-ethyl extraction at 1, 3, 5, 7 and 9 days after treatment. The experiment was conducted with three replicates.

The samples collected were extracted according to the methods of Mei et al. with modification [48], then transferred to the 10 mL centrifuge tubes (Biosharp, Guangzhou, China). After adding 5 mL methyl alcohol (Sigma, Beijing, China), the homogenate was sonicated for 30 min. Before the homogenate was centrifuged (3904× *g*, 5 min), 1 g NaCl was added and vortexed violently for 2 min. The supernatant was blown by nitrogen at 40 °C, and dissolved in 0.5 mL methyl alcohol. After centrifugation at 10,844× *g* for 5 min, 400 μL of the supernatant was filtered by 0.22 µm filter (Biosharp, Guangzhou, China) and detected by high-performance liquid chromatography (HPLC). Chromatographic separation was performed as described [20]. The 20 μL aliquot was injected and the mobile phase was composed of 70% methyl alcohol and 30% ultrapure water (*v*/*v*) containing 0.1% acetic acid.

Linearity was calculated using quizalofop-p-ethyl standard substance at concentration of 0.1, 0.5, 1, 5, 10, 20 mg·L^−1^. Precision was calculated as a relative standard deviation (RSD) from recovery tests with standard solution (*n* = 3) at levels of 1, 5, and 10 mg·L^−1^.

### 4.5. Development of dCAPS for 2078 Mutation Detection and Genotype Analysis

When the plants grew to tillering stage under the conditions described above, a single leaf of each plant was collected for detecting mutation and genotype analysis. In order to realize a quick determination of the mutant Asp allele at the 2078 position of ACCase in *P. fugax*, one pair of primers (2078F: 5′-CCCCAAGGCTGCAGAGCTCCGTGGAGGGGCGTGGGTCGTGACTA-3′ and 2078R: TTCTGGATCAAGCCTACCCAT-3′), was designed using dCAPS Finder (http://helix.wustl.edu/dcaps/ (accessed on 23 January 2022)). The primer 2078F was introduced by two forced mismatches (underlined) to create a restriction site for *SpeI* (Thermo Fisher, Catalog No. ER1251) in the mutant biotypes. Thus, the mutant-type amplicons digested by *SpeI* would generate two bands of 143- and 41-bp. In contrast, the wild-type amplicons would develop a single undigested 184-bp band due to the unavailability. of the *SpeI* restriction site. PCRs were performed in a total volume of 25 μL containing 1 μL gDNA, 1 μL of each primer (10 μM), 12.5 μL of 2 × PCR Green Master Mix (Promega, Madison, WI, USA) and 9.5 μL ddH_2_O. PCRs were run as the following program: 4 min initial denaturation at 94 °C, 35 cycles of 30 s at 94 °C, 30 s at 55 °C, 30 s at 72 °C, and a final extension step of 72 °C for 7 min. The digested and undigested PCR amplicons were separated on 3% agarose gels (Tsingke, Beijing, China).

To confirm the accuracy of this dCAPS method, a total of 32 plants in R population and 2 plants in S population were random selected for the dCAPS detection, and the remaining part was sprayed with quizalofop-p-ethyl at 105 g a.i. ha^−1^ (twice the recommend-field rate). Three weeks after quizalofop-p-ethyl application, their growth condition was observed and confirmed to be either alive or dead.

### 4.6. Statistical Analyses

Through the analysis of ANOVA, no significant difference in the fresh weight data of the repeated experiment was observed using SPSS v23 (IBM, Armonk, NY, USA) [17]. Therefore, we merged all data from the repeated experiment. To determine the herbicide dose causing a 50% reduction of fresh weight (GR_50_), it was estimated by a four-parameter non-linear logistic-regression model using SigmaPlot 13.0 (Systat Software, Inc., San Jose, CA, USA). The fitted model is shown below:

$$y=C+\frac{D-C}{1+{\left(x/GR_{50}\right)}^{b}}$$

where *C* is the lower limit, *D* is the upper limit, b is the slope of *GR*_50_. The ratio of *GR*_50_ value of resistant population and *GR*_50_ value of the sensitive population was used to estimate resistance index (RI).

## 5. Conclusions

Investigation of resistance mechanisms and cross-resistance patterns in herbicide-resistant weeds can help develop more effective weed management methods [40,46]. The quizalofop-p-ethyl-resistant *P. fugax* has evolved a cross-resistance to nine ACCase inhibitors. This will lead to the rising of herbicide resistant *P. fugax* populations in China. Fortunately, two ALS-inhibitors tested were still able to control this R population. However, for the control of weeds with this resistance mechanism in canola fields, there is an urgent need to improve the diversity of weed management practices rather than solely relying on herbicides.

## Figures and Tables

**Table 1:** Effects of cytochrome P450 inhibitors (malathion and PBO) and GST inhibitor (NBD-Cl) on *Polypogon fugax* growth response to quizalofop-p-ethyl.

Population	Treatment †	GR_50_ (g a.i.ha^−1^) (SE) ^a^	RI
R	Q	102.05 (1.19)	10.89
	Q + N	95.41 (1.31)	10.18
	Q + M	96.86 (1.37)	10.34
	Q + P	100.20 (3.43)	10.69
S	Q	9.37 (0.22)	1.00
	Q + N	9.59 (0.56)	1.02
	Q + M	8.80 (0.23)	0.94
	Q + P	9.85 (0.70)	1.05

^a^ SE, standard error; GR_50_, the herbicide dose causing 50% reduction of fresh weight; RI, resistance index defined as the ratio of GR50 value of resistant population and GR50 value of sensitive population. R, the resistant population; S, the sensitive population. † Q, quizalofop-p-ethyl; N, NBD-CI; M, malathion; P, PBO.

**Table 2:** Sensitivities of the quizalofop-p-ethyl-resistant and -susceptible *Polypogon fugax* populations to other acetyl-CoA carboxylase inhibitors and acetolactate synthase inhibitors.

Herbicide	Population	GR_50_ (g a.i.ha^−1^) (SE) ^a^	RI
Clodinafop-propargyl	R	42.73 (0.20)	9.07
	S	4.71 (0.13)	
Haloxyfop-R-methyl	R	48.20 (3.55)	13.65
	S	3.53 (0.44)	
Sethoxydim	R	72.15 (3.79)	8.82
	S	8.18 (1.60)	
Pinoxaden	R	33.88 (2.62)	5.46
	S	6.20 (0.66)	
Fenoxaprop-P-ethyl	R	97.53 (2.68)	18.40
	S	5.30 (0.50)	
Metamifop	R	156.58 (4.18)	10.77
	S	14.54 (1.17)	
Clethodim	R	23.36 (0.39)	6.54
	S	3.57 (0.12)	
cyhalofop-butyl	R	114.82 (4.27)	10.00
	S	11.48 (1.89)	
Mesosulfuron-methyl	R	9.54 (2.45)	1.31
	S	7.27 (1.24)	
pyroxsulam	R	7.82 (2.55)	0.88
	S	8.91 (2.67)	

^a^ SE, standard error; GR_50_, the herbicide dose causing 50% reduction of fresh weight; RI, resistance index defined as the ratio of GR_50_ value of resistant population and GR_50_ value of sensitive population. R, the resistant population; S, the sensitive population.

**Table 3:** Dose-response to acetyl-CoA carboxylase inhibitors and acetolactate synthase inhibitors.

	Group †	Herbicide	Formulation ‡	Company	Treated Doses (g a.i. ha^−1^) ^a^
ACCase	APP	quizalofop-p-ethyl Clodinafop-propargyl	10% EC 15% ME	Tianrun Hansi	0, 6.56, 13.13, 26.25, 52.5, 105, 210 0, 5.63, 11.25, 22.5, 45, 90, 180
		Haloxyfop-R-methyl	108 g L^−1^ EC	Flag	0, 2, 4, 8, 16, 32, 64
		Fenoxaprop-P-ethyl	69 g L^−1^ EW	Bayer	0, 7.76, 15.53, 31.05, 62.1, 124.2, 248.4
		Metamifop	10% EC	FMC	0, 12.5, 25,50, 100, 200, 400
		cyhalofop-butyl	100 g L^−1^ EC	Jiahui	0, 12.5, 25,50, 100, 200, 400
	CHD	Clethodim	240 g L^−1^ EC	Cynda	0, 3.1, 6.1, 12.2, 24.3, 48.6, 97.2
		Sethoxydim	12.5% EC	Cynda	0, 19.5, 39, 78, 156, 312, 624
	DEN	Pinoxaden	5% EC	Syngenta	0, 5.6, 11.3, 22.5, 45, 90, 180
ALS		Mesosulfuron-methyl	30 g L^−1^ OD	FMC	0, 1.41, 2.81, 5.63, 11.25, 22.5, 45
		pyroxsulam	4% OD	Dow	0, 1.13, 2.25, 4.5, 9, 18, 36

† APP, aryloxyphenoxypropionate; CHD, cyclohexanedione; DEN, phenylpyraxoline. ‡ EC, emulsifiable concentrate; ME, microemulsion; EW, emulsion in water; OD, oil dispersion. ^a^ The recommended field rate was underlined.

## Data Availability

Not applicable.

## Supplementary Materials

The supporting information can be downloaded at: https://www.mdpi.com/article/10.3390/ijms24010528/s1.
