# Supplementary material for: First Asp-2078-Gly Mutation Conferring Resistance to Different ACCase Inhibitors in a Polypogon fugax Population from China
Source: Int J Mol Sci. 2022 Dec 28;24(1):528. doi: 10.3390/ijms24010528 (PMC9820770; doi:10.3390/ijms24010528)
Supplement: Supplementary file 1 [file ijms-24-00528-s001.zip › ijms-2083786-supplementary.pdf]

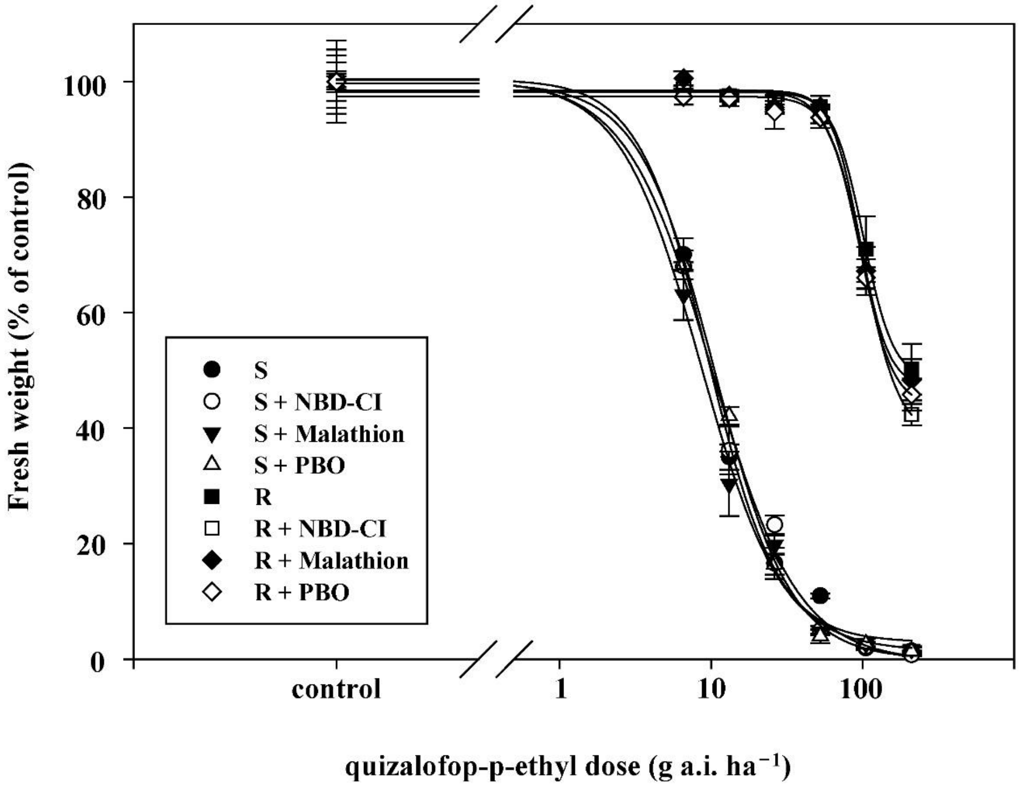

Figure S1. Sensitivities of the R and S *Polypogon fugax* populations to quizalofop-P-ethyl, quizalofop-P-ethyl plus NBD-Cl, quizalofop-P-ethyl plus malathion or quizalofop-P-ethyl plus PBO. R, the resistant population; S, the sensitive population; NBD-Cl, 4-chloro7-nitrobenzoxadiazole; PBO, piperonyl butoxide.

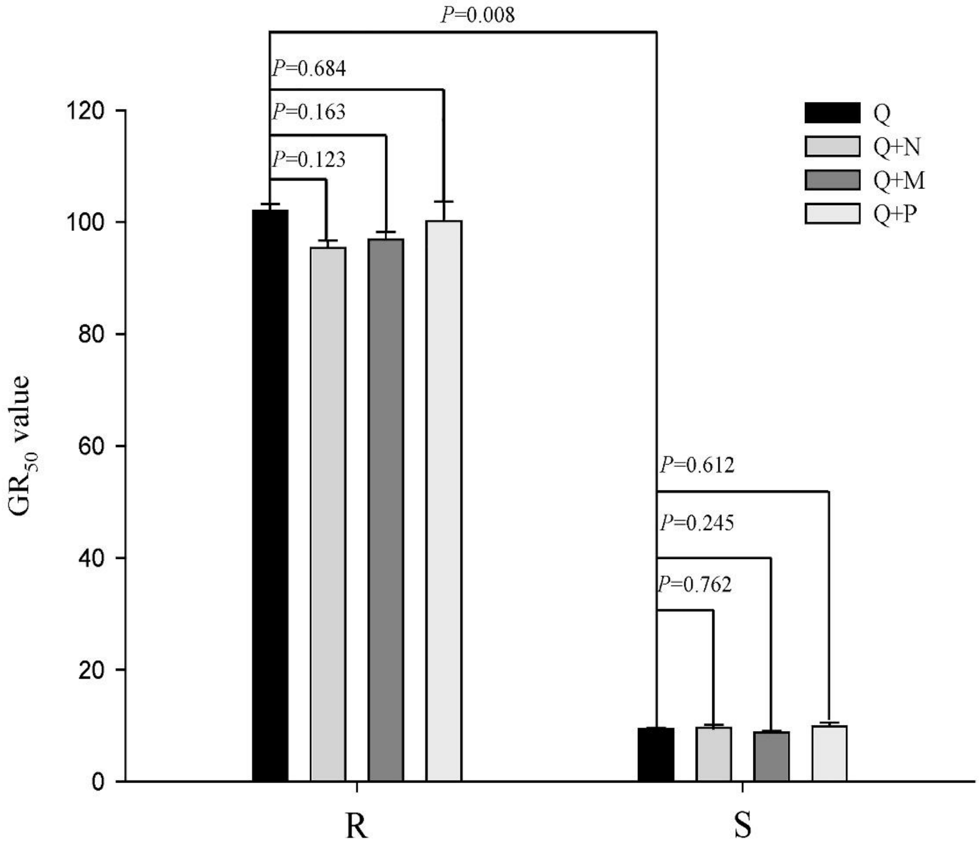

Figure S2. The significant difference of GR_{50} values between the two runs in different quizafop-p-ethyl treatment groups. R, the resistant population; S, the sensitive population. Q, quizalofop-p-ethyl; N, NBD-CI; M, malathion; P, PBO. GR_{50}, the herbicide dose causing 50% reduction of fresh weight; Mean comparison was performed using the single sample *t-test* by SPSS v23 (IBM, Armonk, USA).

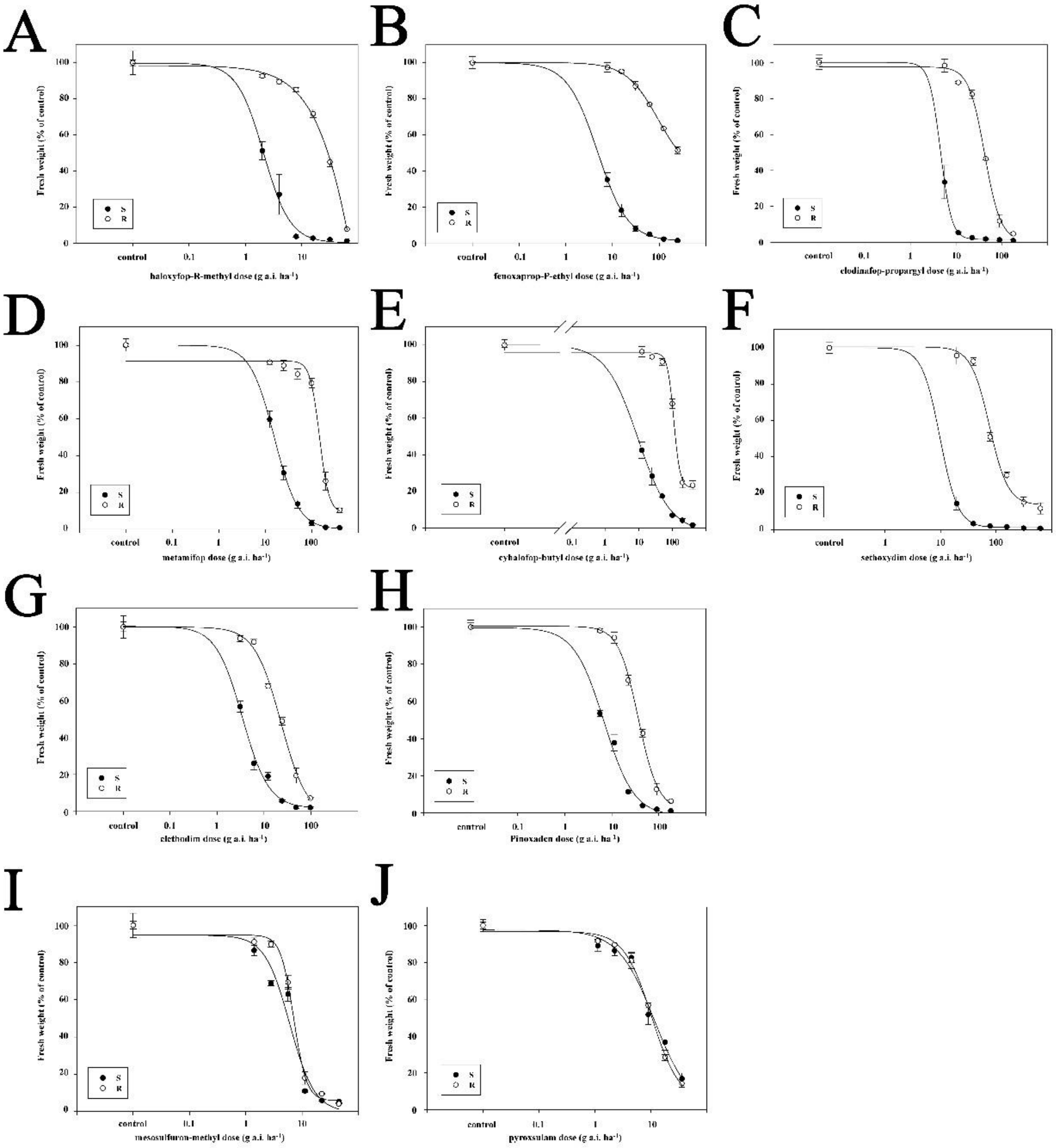

Figure S3. Growth response of the quizalofop-p-ethyl-resistant and -susceptible *Polypogon fugax* populations to other acetyl-CoA carboxylase inhibitors and acetolactate synthase inhibitors. R. the resistant population; S, the sensitive population;Acetyl-CoA carboxylase inhibitor: A, haloxyfop-R-methyl; B, fenoxaprop-P-ethyl; C, clodinafop-propargyl; D, metamifop; F, cyhalofop-butyl; G, sethoxydim; H, clethodim; I, pinoxaden. Acetolactate synthase inhibitor: I, mesosulfuron-methyl; J, pyroxsulam.

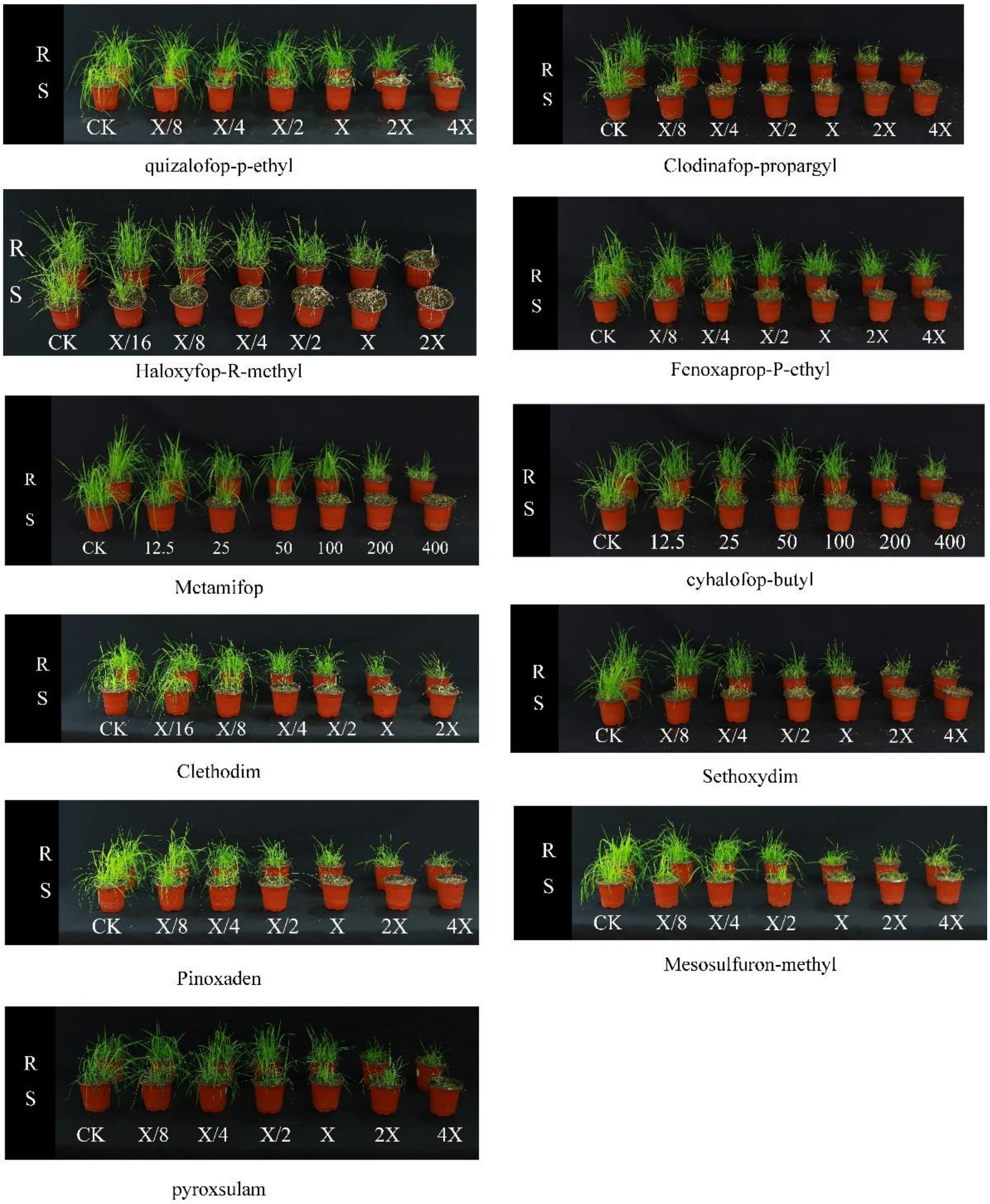

Figure S4. Growth effects of each inhibitor on the resistant and susceptible Polypogon fugax populations. R, the resistant population; S, the sensitive population; X, the recommended field rate.

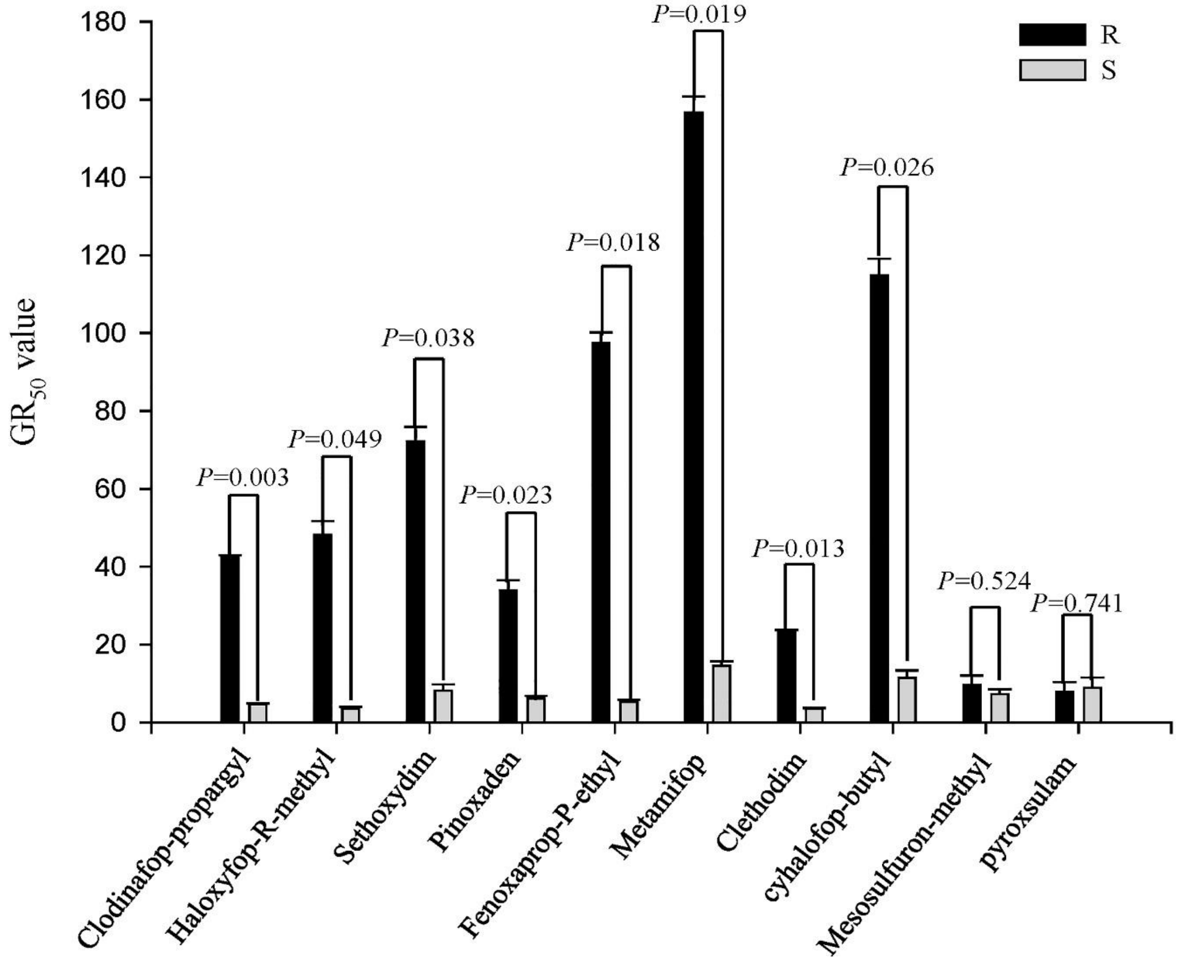

Figure S5. The significant difference of GR_{50} values between the two runs in different herbicides. R, the resistant population; S, the sensitive population. GR_{50}, the herbicide dose causing 50% reduction of fresh weight; Mean comparison was per-formed using the single sample t-test by SPSS v23 (IBM, Armonk, USA).

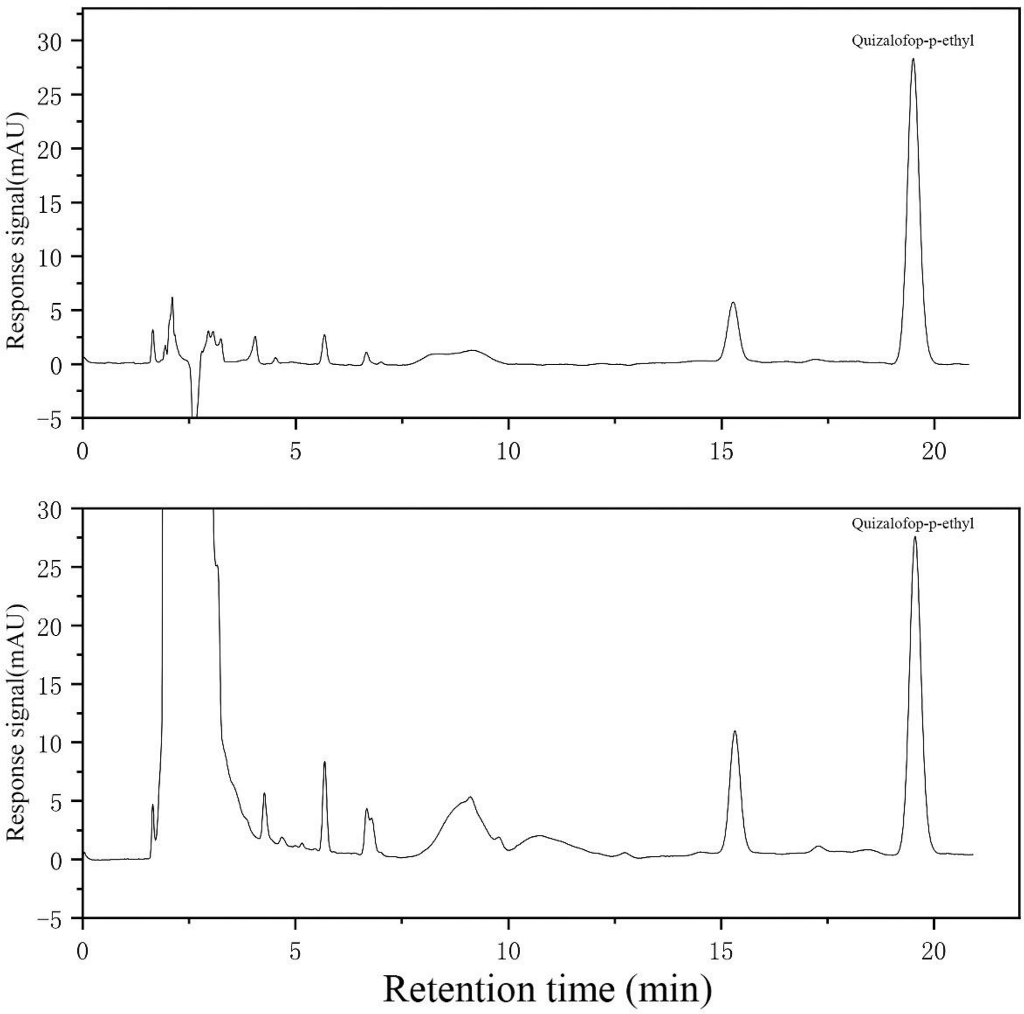

Figure S6. Typical chromatograms of quizalofop-P-ethyl from standard (up) and extract samples (down).
